# Impact of Preoperative Low Prognostic Nutritional Index and High Intramuscular Adipose Tissue Content on Outcomes of Patients with Oral Squamous Cell Carcinoma

**DOI:** 10.3390/cancers12113167

**Published:** 2020-10-28

**Authors:** Takuya Yoshimura, Hajime Suzuki, Hirotaka Takayama, Shotaro Higashi, Yuka Hirano, Masahiro Tezuka, Takayuki Ishida, Kiyohide Ishihata, Yasuhiro Nishi, Yasunori Nakamura, Yasushi Imamura, Etsuro Nozoe, Norifumi Nakamura

**Affiliations:** 1 Department of Oral and Maxillofacial Surgery, Kagoshima University Graduate School of Medical and Dental Sciences, Kagoshima 890-8520, Japan; y-taku@dent.kagoshima-u.ac.jp (T.Y.); h-tkym@d1.dent.kagoshima-u.ac.jp (H.T.); higashi@d1.dent.kagoshima-u.ac.jp (S.H.); y-hrn@d1.dent.kagoshima-u.ac.jp (Y.H.); tetsu@dent.kagoshima-u.ac.jp (M.T.); taka-isi@dent.kagoshima-u.ac.jp (T.I.); ishihata@dent.kagoshima-u.ac.jp (K.I.); nozoe@dent.kagoshima-u.ac.jp (E.N.); nakamura@dent.kagoshima-u.ac.jp (N.N.); 2 Department of Oral and Maxillofacial Prosthodontics, Kagoshima University Graduate School of Medical and Dental Sciences, Kagoshima 890-8520, Japan; shar@dent.kagoshima-u.ac.jp; 3 Department of Oral Surgery, National Hospital Organization Kagoshima Medical Center, Kagoshima 892-0853, Japan; nakamura.yasunori.mc@mail.hosp.go.jp; 4 Department of Internal Medicine, Kagoshima Kouseiren Hospital, Kagoshima 890-0062, Japan; yasushii@po.synapse.ne.jp

**Keywords:** oral squamous cell carcinoma, sarcopenia, computed tomography, psoas muscle mass index, intramuscular adipose tissue content

## Abstract

**Simple Summary:**

Oral squamous cell carcinoma (OSCC) has the highest mortality rate among all head and neck cancers. To date, the impact of preoperative malnutrition and sarcopenia on survival in OSCC patients remains controversial. The aim of our study was to investigate the effects of preoperative nutritional status and abnormalities in body composition on the mortality of OSCC patients. The disease-specific survival (DSS) rate among patients with a high intramuscular adipose tissue content (IMAC) and a low psoas muscle mass index was significantly lower than that in controls. Multivariate analysis revealed that a low preoperative Prognostic Nutritional Index (PNI) and high IMAC were independent risk factors. We demonstrated that preoperative malnutrition and abnormal body composition, such as abnormal preoperative skeletal muscle quality, are associated with DSS in OSCC patients. Our study suggests that the evaluation of preoperative malnutrition and skeletal muscle quality would be useful for predicting mortality in patients with OSCC.

**Abstract:**

The impact of preoperative malnutrition and sarcopenia on survival in oral squamous cell carcinoma (OSCC) patients remains controversial. We investigated the effects of the preoperative nutritional status and abnormalities in body composition on the mortality of OSCC patients. A retrospective study involving 103 patients with OSCC was conducted. Disease-specific survival (DSS) according to the preoperative psoas muscle mass index (PMI) and intramuscular adipose tissue content (IMAC) was evaluated. Univariate and multivariate analyses were performed to determine the predictive performance of the covariates with respect to DSS. The DSS rate in patients with high IMAC and low PMI was significantly lower than that in controls. Multivariate analysis revealed that a low preoperative Prognostic Nutritional Index (PNI) and high IMAC were independent risk factors. We demonstrated that preoperative malnutrition and abnormal body composition, such as preoperative skeletal muscle quality, are associated with DSS in OSCC patients. Our study suggests that the evaluation of preoperative malnutrition and skeletal muscle quality would be useful for predicting mortality in patients with OSCC.

## 1. Introduction

Oral squamous cell carcinoma (OSCC) has the highest mortality rate among all head and neck cancers (HNCs) [1], even though advances in treatment have improved survival rates. Recently, the global estimates have revealed 354,864 new cases and 177,384 deaths in 2018, which ranks OSCC sixth in terms of incidence rate among malignant tumors worldwide [1,2]. The incidence of OSCC has increased in many countries, especially in younger individuals, but whether young and old patients with OSCC have a different prognosis is controversial [2]. Recent studies have revealed no significant differences in tumor stage or grade in the comparisons of the characteristics of OSCC in young and old patients [3,4,5]. Therefore, the discovery of factors that facilitate OSCC prognosis is one of the key challenges that we need to overcome.

A previous report described that up to 46–49% of patients with HNC have a significantly higher risk of severe malnutrition and subsequent sarcopenia than patients with other malignancies [6]. The Prognostic Nutritional Index (PNI), which is defined according to the combined parameters of albumin and lymphocytes, may be particularly useful due to its role as a surrogate marker of both inflammation and nutritional status, which reflect acute inflammation and malnutrition, respectively [7]. A low PNI has been associated with severe radiotherapy-induced adverse events in patients with HNC [8]. In addition, a retrospective cohort study suggested that PNI was an independent prognostic biomarker for the overall survival of patients with locally advanced head and neck squamous cell carcinoma [7].

In recent years, sarcopenia has emerged as a negative prognostic factor not only in old patients but also in cancer patients [9]. The comorbidity of sarcopenia in cancer patients leads to a higher rate of postoperative complications, longer hospital stays and decreased survival after surgery [9]. Optimal care for individuals with sarcopenia is mandatory because the condition can lead to high personal, social and economic burdens when left untreated [10]. According to the operational definition of sarcopenia provided by the European Working Group on Sarcopenia in Older People 2 (EWGSOP2), a sarcopenia diagnosis is confirmed by the presence of low muscle quality or quantity [11]. Muscle quality is a relatively new term that refers to both microscopic and macroscopic changes in muscle architecture and composition as well as to muscle function delivered per unit of muscle mass [11]. As yet, no universal consensus on the assessment methods in routine clinical practice has been established [11], but advanced diagnostic tools such as MRI and CT have been used to estimate muscle quality in research settings, e.g., by determining infiltration of fat into muscle, the attenuation of the muscle and the volume of intermuscular adipose tissue [12,13]. Muscle quantity can be reported as total body skeletal muscle mass, appendicular skeletal muscle mass, or the cross-sectional area of specific muscle groups or body locations, but it remains controversial as to which method is better [11]. In particular, CT-based measurements of the psoas muscle have been reported to be simple and predictive of morbidities in various status, such as cirrhosis and locally advanced gastric cancer, as well as in patients who undergo colorectal surgery [14].

Several lines of evidence indicate that sarcopenic obesity, defined as the combination of low muscle mass and BMI, is an independent risk factor for death after resection of hepatocellular carcinoma or pancreatic cancer [15,16]. Kamo et al. showed that patients with sarcopenic obesity exhibited worse survival after living donor liver transplantation compared with nonsarcopenic/nonobese patients [17]. In contrast, the impact of preoperative sarcopenic obesity on the outcomes of patients with OSCC is still not completely understood.

To date, no reports have directly discussed the impact of the preoperative comorbidity of sarcopenia on OSCC patients. Consequently, the correlation between preoperative sarcopenia and the mortality of OSCC patients remains unclear. Previous studies have focused on the intramuscular adipose tissue content (IMAC) as the quality of skeletal muscle mass and the psoas muscle mass index (PMI) as the quantity of skeletal muscle mass [18,19]. Therefore, in this study, we investigated the effects of preoperative nutritional status and abnormalities in body composition, which reflect sarcopenic conditions, such as high IMAC and low PMI, on the mortality of patients with OSCC.

## 2. Materials and Methods

### 2.1. Patients

A retrospective cohort study was conducted and included 113 patients with primary OSCC who underwent surgical treatment at our institution between January 2009 and December 2015. No patient had distant metastasis at the time of diagnosis. We excluded 10 of these patients from the analysis, as they did not undergo a preoperative ^18^F-fluorodeoxyglucose positron emission tomography/computed tomography (FDG PET/CT) scan. Therefore, this study enrolled and evaluated the images of 103 patients (61 men, 42 women). All patients were followed up from surgery for a minimum of 4 years or until death. This study complied with the standards of the Declaration of Helsinki and the current ethical guidelines and was approved by the Kagoshima University Ethics Committee (approval No. 29-14).

### 2.2. Image Analysis

**Figure 1:**
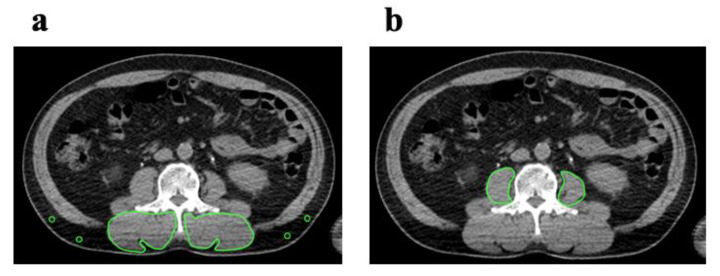
Cross-sectional CT images at the L3 level. (**a**) IMAC was evaluated by dividing the CT value of bilateral multifidus muscles according to the CT attenuation value for subcutaneous fat. (**b**) Subfascial muscle tissue in the multifidus muscle was precisely traced. The PMI was calculated by normalizing the cross-sectional areas to height (cm^2^/m^2^). CT, computed tomography; IMAC, intramuscular adipose tissue content; PMI, psoas muscle index.

We used the CT component of FDG PET/CT images as a single measure of whole-body imaging to detect primary squamous cell carcinoma 7–14 days before surgery. Cross-sectional areas (cm^2^) of skeletal muscle in the third lumbar vertebrae (L3) region and CT values (in Hounsfield units) were both measured with OsiriX v.4.0 (Pixmeo SARL, Geneva, Switzerland). Intramuscular adipose tissue content (IMAC) and PMI were calculated according to a previously reported procedure [20]. Manual tracing using preoperative CT imaging at the L3 level was used to measure the cross-sectional areas of the right and left psoas muscles. Skeletal muscle areas are calculated as areas of −29 to 150 HU. Visceral and subcutaneous adipose tissue areas are calculated as areas of −150 to −50 HU. IMAC was calculated as the region of interest (ROI) of the multifidus muscle (Hounsfield units)/ROI of subcutaneous fat (Hounsfield units). CT values were measured for four circular ROIs on subcutaneous fat away from major vessels. The mean values of these 4 ROIs were used as the ROI of subcutaneous fat (Figure 1a). The PMI was calculated by normalizing the cross-sectional areas to height (cm^2^/m^2^).

### 2.3. Cut off Values of PMI and IMAC

Since the ranges of PMI and IMAC in men and women are quite different [15,16], we calculated different cutoff lines for each using receiver operating characteristic (ROC) curves. Then, the cutoff values for the optimal estimation of PMI and IMAC in males and females were determined using the Youden Index. The cutoff values for the PMI in males and females were 6.05 (area under the curve [AUC] = 0.7193, *p* = 0.1452) and 5.097 (AUC = 0.6111, *p* = 0.3883), respectively. On the basis of these cutoff values, patients were assigned to one of two groups, low PMI or normal PMI. The cutoff values for IMAC in males and females were −0.3352 (AUC = 0.5877, *p* = 0.5601) and 0.06 (AUC = 0.7685, *p* = 0.0371), respectively. On the basis of these cutoff values, patients were assigned to one of two groups, high IMAC or normal IMAC.

### 2.4. Analyzed Parameters

The following clinicopathological variables were collected for all patients: demographics (sex and age), BMI (kg/m^2^), comorbidities, preoperative PNI (used as continuous variables), tumor location, TNM classification, tumor stage (according to the American Joint Committee on Cancer [AJCC] Cancer Staging Manual, Seventh Edition), treatment and survival outcomes. PNI was calculated as 10 * albumin (g/dL) + 0.005 * total lymphocyte count (per μL). Disease-specific survival (DSS) and overall survival (OS) according to preoperative PMI and IMAC were evaluated using the log-rank test or the Mantel–Cox test. A univariate analysis was performed using the log-rank test to assess the differences in survival with respect to certain covariates. A multivariate analysis was performed using Cox proportional hazards regression models to determine the predictive performance of covariates with respect to DSS and was reported as the hazard ratio (HR) with a 95% confidence interval (CI).

A BMI ≥ 25 kg/m^2^ indicated obesity according to the guidelines of the Japan Society for the Study of Obesity [21]. This study defined sarcopenic obesity as the combination of low PMI (males, <6.05 cm^2^/m^2^; females, <5.097 cm^2^/m^2^) and a BMI ≥ 25 kg/m^2^. Based on these definitions, we divided all patients into the nonsarcopenic/non-obesity (NN), nonsarcopenic/obesity (NO), sarcopenic/non-obesity (SN) and sarcopenic/obesity (SO) groups according to PMI and BMI and in accordance with a recent report [17]. The characteristics of each groups are summarized in Table S1. Cumulative DSS and OS rates were calculated by the Kaplan–Meier method and differences between curves were evaluated by the log-rank test.

### 2.5. Statistical Analysis

Statistical significance was set at *p* < 0.05. Any variable identified as significant (*p* < 0.05) or that showed a value of *p* < 0.10 in the univariate analyses was considered a candidate for the multivariate Cox proportional hazards model. All statistical analyses were performed using GraphPad Prism version 6.0 for Mac OS X (GraphPad Software, San Diego, CA, USA) and Stata version 16 (StataCorp LLC, College Station, TX, USA).

## 3. Results

The patient demographics are summarized in Table 1.

**Figure 2:**
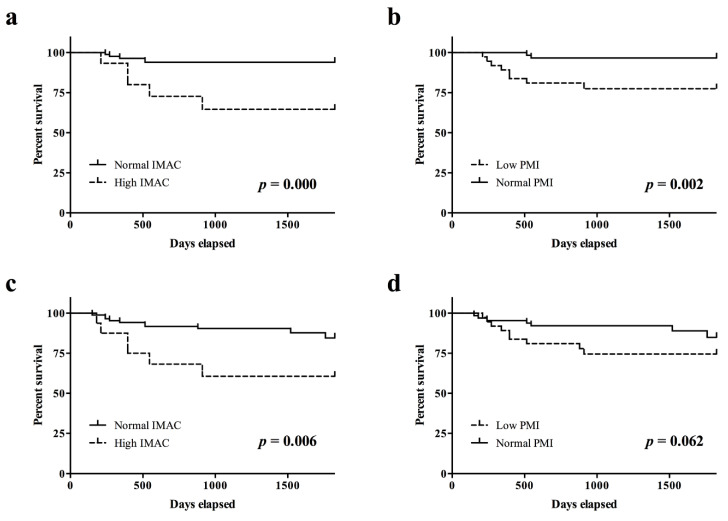
Survival rates according to preoperative IMAC and PMI. (**a**) The disease-specific survival rate in patients with a high IMAC was significantly lower than that in patients with a normal IMAC (*p* = 0.000). (**b**) The disease-specific survival rate in patients with a low PMI was significantly lower than that in patients with a normal PMI (*p* = 0.002). (**c**) The overall survival rate in patients with a high IMAC was significantly lower than that in patients with a normal IMAC (*p* = 0.006). (**d**) The overall survival rate in patients with a low PMI trended toward lower than that in patients with a normal PMI (*p* = 0.062). IMAC, intramuscular adipose tissue content; PMI, psoas muscle mass index.

The DSS rate in patients with high IMAC was significantly lower than that in patients with normal IMAC (*p* < 0.001; Figure 2a). The DSS rate in patients with a low PMI was significantly lower than that in patients with a normal PMI (*p* < 0.005; Figure 2b). The OS rate in patients with a high IMAC was significantly lower than that in patients with a normal IMAC (*p* < 0.01; Figure 2c). The OS rate in patients with a low PMI trended toward lower than that in patients with a normal PMI (Figure 2d).

In the univariate analysis, the following covariates were associated with a statistically significant difference in DSS: PNI (*p* = 0.021), IMAC (*p* = 0.006) and PMI (*p* = 0.011). According to the multivariate Cox proportional hazards regression, a low PNI was associated with shorter DSS (HR, 0.856; 95% CI 0.755–0.970). High IMAC was associated with shorter DSS (HR, 6.159; 95% CI 1.773–21.395). The corresponding HRs, CIs and *p* values are summarized in Table 2.

No significant differences in DSS rates were observed among the four groups of NN, NO, SN and SO (*p* = 0.9501, Figure S1).

## 4. Discussion

Various lines of evidence show that both malnutrition and sarcopenia are predictive of mortality for a variety of diseases. The number of reports related to body composition assessment for the purpose of nutritional evaluation and prognostication in patients with cancer has increased. In the current retrospective study, preoperative abnormal body composition, such as low PMI and high IMAC, were significantly related to DSS. These results suggest a positive correlation between the preoperative comorbidity of sarcopenia and the mortality of OSCC patients. Additionally, we demonstrated that the prevalence of preoperative low PNI and high IMAC were independent predictors of survival in OSCC patients. The present study revealed the potential effect of both malnutrition and sarcopenia on survival in OSCC.

In the current retrospective study, we showed that high IMAC, which represents the prevalence of low muscle quality, was an independent predictor of mortality in OSCC patients in the multivariate analysis. IMAC was calculated by dividing the CT value of the multifidus muscles with that of the subcutaneous fat [20,22]. Since the value of IMAC is normalized to the value of subcutaneous fat in each patient, it is not affected by the CT system or scanning conditions [20]. Therefore, it can more sensitively reflect skeletal muscle quality [23]. The underlying mechanism of the results remains unclear, but accumulating evidence has indicated that skeletal muscle loss associated with increasing adipose tissue leads to the synthesis and secretion of various proinflammatory adipokines including leptin, tumor necrosis factor alpha, interleukin (IL)-1 and IL-6 [24]. On the contrary, decreases in adiponectin or myokines, including decreases in IL-15 levels, in the sarcopenic population had inhibitory effects on the immune system, particularly with respect to natural killer lymphocytes in the innate immune system [19,25]. Further research is needed to confirm the detailed mechanism.

In our study, muscle quantity as defined by the PMI was statistically significant in the univariate analysis but not in the multivariate analysis. Therefore, we cannot conclude that PMI is a risk factor for survival in OSCC. However, a preoperative depletion of muscle quantity would be a risk factor for sarcopenia in OSCC because it accounts for the main pathology of sarcopenia. Recently, McSharry et al. [26] conducted a systematic review and meta-analysis on the prognostic potential of sarcopenia in the context of the skeletal muscle index (SMI) and low muscle attenuation in relation to the three- and five-year survival rates (3YSRs and 5YSRs) of patients with epithelial ovarian cancer (EOC). Interestingly, normal muscle loss was significantly associated with improved 3YSRs and 5YSRs in patients with EOC, while low SMI was not significantly associated with either 3YSR or 5YSR in patients with EOC [26]. The authors presumed that sarcopenia was assessed at one time point, was not a measure of actual muscle loss and may be influenced by a patient’s innate muscularity [26]. A recent study that focused on psoas muscle area (PMA) measurements rather than measurement of the entire SMI at the L3 lumbar vertebra level (L3SMI) showed that PMA provided better accuracy than L3SMI [27]. On the other hand, other experts argue that the psoas muscle is not representative of overall sarcopenia because it is a minor muscle [28,29,30]. In practice, measuring the PMA rather than measuring all skeletal muscle areas is simpler and faster and is most likely more informative because ascites and associated abdominal wall edema may reduce the accuracy of peripheral abdominal skeletal muscle measurements [31].

A previous report assessed the prevalence and clinical implications of sarcopenic obesity in cancer patients and demonstrated great variability in body composition and links to clinical implications, including functional status, survival and chemotherapy toxicity [32]. Recent studies have shown the positive relationship between low skeletal muscle mass and low BMI and surgical site infection in free flap reconstruction for oral cancer [33,34]. In our current retrospective study, BMI was not an independent prognostic factor in patients with OSCC according to the multivariate analysis. Additionally, no significant differences were apparent in patients with OSCC between the NN, NO, SN and SO groups, which may be because the number of patients in the SO group was too small (*n* = 5). A further prospective study to investigate the impact of sarcopenic obesity on outcomes of patients with OSCC is needed.

In our retrospective study, there was a discrepancy in that tumor size and lymph node involvement were not associated with survival. Additionally, we found that there were no statistically significant differences between the groups in DSS or OS according to T and N classification (Figure S2). The typical prognostic factors after cancer treatment are clearly based on tumor stage, surgical pathology and lymph node involvement. However, in accordance with a recent study, postoperative survival is a complex process not necessarily related to tumor characteristics [35]. Longer and larger prospective studies are needed to better understand these results.

The current study has some limitations. First, this study was retrospective in nature and had a relatively small sample size with an unequal distribution of males and females. Therefore, the selection bias of patients could not be fully excluded. A further prospective, randomized controlled study is therefore needed to confirm our findings. Second, the cutoff values for IMAC and PMI are not yet well defined to identify and characterize sarcopenia. However, the definition of sarcopenia has not been established and there is no definitive method or specific numerical thresholds [36]. EWGSOP2 recommends simple, specific cutoff points for measurement [11], but these cutoff values would most likely differ from those calculated in Asian populations due to variances in body size, lifestyles and ethnicities [37,38]. The cutoff values for IMAC and PMI in our study were similar to those reported in a previous paper [18]. Previous studies indicate that PMI has comprehensive cutoff values for low muscle mass calculated from healthy adults [39]. The validity of the proposed cutoff values will need to be confirmed in prospective longitudinal intervention studies.

## 5. Conclusions

We demonstrated that preoperative malnutrition and abnormal body composition, such as abnormal preoperative skeletal muscle quality, are associated with DSS in patients with OSCC. Therefore, evaluation of preoperative malnutrition and skeletal muscle quality and quantity using the proper methods would be useful for predicting the mortality and optimizing the treatment of OSCC patients. Additionally, aggressive perioperative nutritional therapy and rehabilitation could be a new strategy to achieve better outcomes.

## Figures and Tables

**Table 1:** Patient characteristics.

Characteristics	*n* = 103
Sex (male/female)	61/42
Age (years)	68 [59–77]
BMI (kg/m^2^)	23.0 [20.5–25.1]
Comorbidities, yes	85
PNI	50.8 [47.4–55.8]
Tumor location	
Tongue/gingival/oral floor/buccal/palate/lip	47/32/11/7/5/1
Classification	
T1/T2/T3/T4	22/54/15/12
N0/N1/N2	61/19/23
Stage	
I/II/III/IV	18/32/21/32
Treatment	
Surgery only	61
Surgery with RT/CT	42
Disease-specific survival (%)	90.3
Disease-free survival (%)	75.7
Overall survival (%)	84.5
Follow up duration	1248 [880–1825]

Continuous data are presented as the medians (interquartile range [IQR]). BMI, body mass index; PNI, prognostic nutritional index.

**Table 2:** Univariate and multivariate analyses using the Cox proportional hazards regression model.

	Univariate			Multivariate		
	HR	95% CI	*p*-Value	HR	95% CI	*p*-Value
Sex	2.089	0.589–7.405	0.254			
Age	1.056	0.994–1.123	0.075			
BMI	0.879	0.730–1.058	0.174			
PNI	0.864	0.764–0.978	0.021	0.856	0.755–0.970	0.015
Comorbidities, yes	1.889	0.239–14.923	0.546			
Location	0.712	0.426–1.190	0.195			
TNM classification						
T	1.144	0.750–2.800	0.269			
N	0.815	0.359–1.850	0.626			
Stage	1.307	0.731–2.335	0.365			
IMAC						
High	5.753	1.663–19.898	0.006	6.159	1.773–21.395	0.004
PMI						
Low	7.553	1.062–35.599	0.011			

BMI, body mass index; PNI, prognostic nutritional index; IMAC, intramuscular adipose tissue content; PMI, psoas muscle mass index; HR, hazard ratio; CI, confidence interval.

## Supplementary Materials

The following are available online at https://www.mdpi.com/2072-6694/12/11/3167/s1, Figure S1: Disease-specific survival rates in patients with OSCC according to PMI and obesity, Figure S2: Survival rates in patients with OSCC according to T and N classification, Table S1 Clinical characteristics classified by PMI and BMI.

## References

[1] Siegel R.L., Miller K.D., Jemal A. (2018). Cancer statistics. CA. Cancer J. Clin..

[2] Almangush A., Mäkitie A.A., Triantafyllou A., de Bree R., Strojan P., Rinaldo A., Hernandez-Prera J.C., Suárez C., Kowalski L.P., Ferlito A. (2020). Staging and grading of oral squamous cell carcinoma: An update. Oral Oncol..

[3] Chen S., Lin Z., Chen J., Yang A., Zhang Q., Xie C., Zhang X., Yang Z., Chen W., Song M. (2020). Older age is a risk factor associated with poor prognosis of patients with squamous cell carcinoma of the oral cavity. Eur. Arch. Oto-Rhino-Laryngol..

[4] Xu Q., Wang C., Li B., Kim K., Li J., Mao M., Qin L., Li H., Huang X., Xing R. (2019). The impact of age on oral squamous cell carcinoma: A longitudinal cohort study of 2,782 patients. Oral Dis..

[5] Bello I.O., Almangush A., Heikkinen I., Haglund C., Coletta R.D., Kowalski L.P., Mäkitie A.A., Nieminen P., Leivo I., Salo T. (2020). Histological characteristics of early-stage oral tongue cancer in young versus older patients: A multicenter matched-pair analysis. Oral Dis..

[6] Pressoir M., Desné S., Berchery D., Rossignol G., Poiree B., Meslier M., Traversier S., Vittot M., Simon M., Gekiere J.P. (2010). Prevalence, risk factors and clinical implications of malnutrition in french comprehensive cancer centres. Br. J. Cancer.

[7] Bruixola G., Caballero J., Papaccio F., Petrillo A., Iranzo A., Civera M., Moriana M., Bosch N., Maroñas M., González I. (2018). Prognostic Nutritional Index as an independent prognostic factor in locoregionally advanced squamous cell head and neck cancer. ESMO Open.

[8] Kono T., Sakamoto K., Shinden S., Ogawa K. (2017). Pre-therapeutic nutritional assessment for predicting severe adverse events in patients with head and neck cancer treated by radiotherapy. Clin. Nutr..

[9] Bozzetti F. (2017). Forcing the vicious circle: Sarcopenia increases toxicity, decreases response to chemotherapy and worsens with chemotherapy. Ann. Oncol..

[10] Mijnarends D.M., Luiking Y.C., Halfens R.J.G., Evers S.M.A.A., Lenaerts E.L.A., Verlaan S., Wallace M., Schols J.M.G.A., Meijers J.M.M. (2018). Muscle, health and costs: A glance at their relationship. J. Nutr. Health Aging.

[11] Cruz-Jentoft A.J., Bahat G., Bauer J., Boirie Y., Bruyère O., Cederholm T., Cooper C., Landi F., Rolland Y., Sayer A.A. (2019). Sarcopenia: Revised European consensus on definition and diagnosis. Age Ageing.

[12] Cruz-Jentoft A.J., Baeyens J.P., Bauer J.M., Boirie Y., Cederholm T., Landi F., Martin F.C., Michel J.-P., Rolland Y., Schneider S.M. (2010). Sarcopenia: European consensus on definition and diagnosis: Report of the European Working Group on Sarcopenia in Older People. Age Ageing.

[13] Commean P.K., Tuttle L.J., Hastings M.K., Strube M.J., Mueller M.J. (2011). Magnetic resonance imaging measurement reproducibility for calf muscle and adipose tissue volume. J. Magn. Reson. Imag..

[14] Waki Y., Irino T., Makuuchi R., Notsu A., Kamiya S., Tanizawa Y., Bando E., Kawamura T., Terashima M. (2019). Impact of preoperative skeletal muscle quality measurement on long-term survival after curative gastrectomy for locally advanced gastric cancer. World J. Surg..

[15] Okumura S., Kaido T., Hamaguchi Y., Fujimoto Y., Kobayashi A., Iida T., Yagi S., Taura K., Hatano E., Uemoto S. (2016). Impact of the preoperative quantity and quality of skeletal muscle on outcomes after resection of extrahepatic biliary malignancies. Surgery.

[16] Okumura S., Kaido T., Hamaguchi Y., Fujimoto Y., Masui T., Mizumoto M., Hammad A., Mori A., Takaori K., Uemoto S. (2015). Impact of preoperative quality as well as quantity of skeletal muscle on survival after resection of pancreatic cancer. Surgery.

[17] Kamo N., Kaido T., Hamaguchi Y., Okumura S., Kobayashi A., Shirai H., Yao S., Yagi S., Uemoto S. (2019). Impact of sarcopenic obesity on outcomes in patients undergoing living donor liver transplantation. Clin. Nutr..

[18] Hamaguchi Y., Kaido T., Okumura S., Kobayashi A., Hammad A., Tamai Y., Inagaki N., Uemoto S. (2016). Proposal for new diagnostic criteria for low skeletal muscle mass based on computed tomography imaging in Asian adults. Nutrition.

[19] Kaido T. (2016). Selection criteria and current issues in liver transplantation for hepatocellular carcinoma. Liver Cancer.

[20] Kitajima Y., Hyogo H., Sumida Y., Eguchi Y., Ono N., Kuwashiro T., Tanaka K., Takahashi H., Mizuta T., Ozaki I. (2013). Severity of non-alcoholic steatohepatitis is associated with substitution of adipose tissue in skeletal muscle. J. Gastroenterol. Hepatol..

[21] Matsuzawa Y., Nakamura T., Takahashi M., Ryo M., Inoue S., Ikeda Y., Ohno M., Sakata T., Fukagawa K., Saitoh Y. (2002). New criteria for “obesity disease” in Japan. Circ. J..

[22] Marcus R.L., Addison O., Kidde J.P., Dibble L.E., Lastayo P.C. (2010). Skeletal muscle fat infiltration: Impact of age, inactivity, and exercise. J. Nutr. Health Aging.

[23] Kitajima Y., Eguchi Y., Ishibashi E., Nakashita S., Aoki S., Toda S., Mizuta T., Ozaki I., Ono N., Eguchi T. (2010). Age-related fat deposition in multifidus muscle could be a marker for nonalcoholic fatty liver disease. J. Gastroenterol..

[24] Tilg H., Moschen A.R. (2006). Adipocytokines: Mediators linking adipose tissue, inflammation and immunity. Nat. Rev. Immunol..

[25] Lutz C.T., Quinn L.B.S. (2012). Sarcopenia, obesity, and natural killer cell immune senescence in aging: Altered cytokine levels as a common mechanism. Aging (Albany NY).

[26] McSharry V., Mullee A., McCann L., Rogers A.C., McKiernan M., Brennan D.J. (2020). The Impact of Sarcopenia and Low Muscle Attenuation on Overall Survival in Epithelial Ovarian Cancer: A Systematic Review and Meta-analysis. Ann. Surg. Oncol..

[27] Golse N., Bucur P.O., Ciacio O., Pittau G., Sa Cunha A., Adam R., Castaing D., Antonini T., Coilly A., Samuel D. (2017). A new definition of sarcopenia in patients with cirrhosis undergoing liver transplantation. Liver Transplant..

[28] Baracos V.E. (2017). Psoas as a sentinel muscle for sarcopenia: A flawed premise. J. Cachexia. Sarcopenia Muscle.

[29] Rutten I.J.G., Ubachs J., Kruitwagen R.F.P.M., Beets-Tan R.G.H., Olde Damink S.W.M., Van Gorp T. (2017). Psoas muscle area is not representative of total skeletal muscle area in the assessment of sarcopenia in ovarian cancer. J. Cachexia. Sarcopenia Muscle.

[30] Ebadi M., Wang C.W., Lai J.C., Dasarathy S., Kappus M.R., Dunn M.A., Carey E.J., Montano-Loza A.J. (2018). Poor performance of psoas muscle index for identification of patients with higher waitlist mortality risk in cirrhosis. J. Cachexia. Sarcopenia Muscle.

[31] Nam N.H., Kaido T., Uemoto S. (2019). Assessment and significance of sarcopenia in liver transplantation. Clin. Transplant..

[32] Prado C.M., Lieffers J.R., McCargar L.J., Reiman T., Sawyer M.B., Martin L., Baracos V.E. (2008). Prevalence and clinical implications of sarcopenic obesity in patients with solid tumours of the respiratory and gastrointestinal tracts: A population-based study. Lancet Oncol..

[33] Nakamura H., Makiguchi T., Yamaguchi T., Suzuki K., Yokoo S. (2020). Impact of sarcopenia on postoperative surgical site infections in patients undergoing flap reconstruction for oral cancer. Int. J. Oral Maxillofac. Surg..

[34] Makiguchi T., Yamaguchi T., Nakamura H., Suzuki K., Harimoto N., Shirabe K., Yokoo S. (2019). Impact of skeletal muscle mass volume on surgical site infection in free flap reconstruction for oral cancer. Microsurgery.

[35] Peyton C.C., Heavner M.G., Rague J.T., Krane L.S., Hemal A.K. (2016). Does sarcopenia impact complications and overall survival in patients undergoing radical nephrectomy for stage III and IV kidney cancer?. J. Endourol..

[36] Kurumisawa S., Kawahito K. (2019). The psoas muscle index as a predictor of long-term survival after cardiac surgery for hemodialysis-dependent patients. J. Artif. Organs.

[37] Harimoto N., Shirabe K., Yamashita Y.I., Ikegami T., Yoshizumi T., Soejima Y., Ikeda T., Maehara Y., Nishie A., Yamanaka T. (2013). Sarcopenia as a predictor of prognosis in patients following hepatectomy for hepatocellular carcinoma. Br. J. Surg..

[38] Fukushima H., Yokoyama M., Nakanishi Y., Tobisu K.I., Koga F. (2015). Sarcopenia as a prognostic biomarker of advanced urothelial carcinoma. PLoS ONE.

[39] Hamaguchi Y., Kaido T., Okumura S., Fujimoto Y., Ogawa K., Mori A., Hammad A., Tamai Y., Inagaki N., Uemoto S. (2014). Impact of quality as well as quantity of skeletal muscle on outcomes after liver transplantation. Liver Transplant..

