# Supplementary material for: Impact of Preoperative Low Prognostic Nutritional Index and High Intramuscular Adipose Tissue Content on Outcomes of Patients with Oral Squamous Cell Carcinoma
Source: Cancers (Basel). 2020 Oct 28;12(11):3167. doi: 10.3390/cancers12113167 (PMC7692578; doi:10.3390/cancers12113167)
Supplement: Supplementary file 1 [file cancers-12-03167-s001.pdf]

## Supplementary Materials

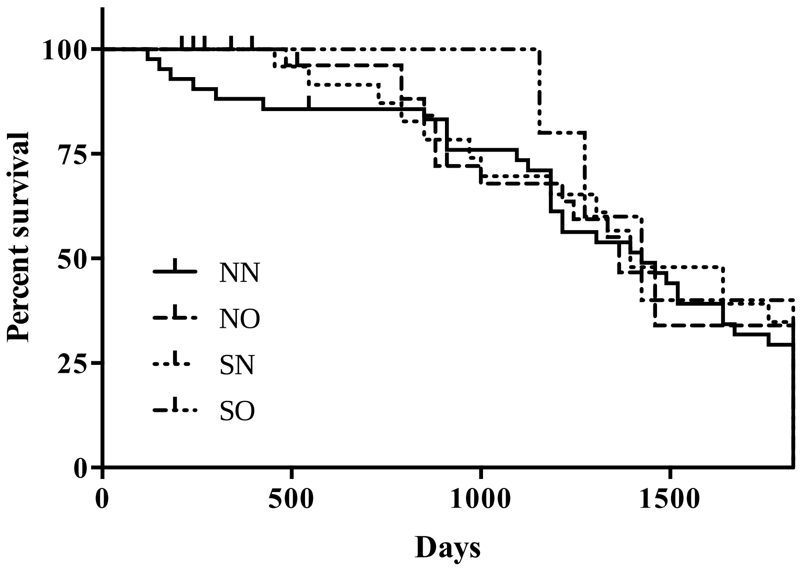


**Figure S1.** Disease-specific survival rates in patients with OSCC according to PMI and obesity. NN, nonsarcopenic/nonobesity; NO, nonsarcopenic/obesity; SN, sarcopenic/nonobesity; SO, sarcopenic/obesity.

**Table S1.** Clinical characteristics classified by PMI and BMI.

| | **NN** (***n***=**42**) | **NO** (***n***=**32**) | **SN** (***n***=**24**) | **SO** (***n***=**5**) |
|---|---|---|---|---|
| Sex (male/female) | 29/13 | 12/20 | 18/6 | 2/3 |
| Age (years) | 63.5 [56–74] | 75 [63.5–81.5] | 69 [60–76] | 67 [62–78] |
| BMI (kg/m$^2$) | 22.1 [20.1–23.5] | 21.4 [19.7–23.0] | 26.9 [25.5–29.1] | 26.8 [26.5–27.5] |
| PNI | 50.5 [46.9–53.4] | 49.8 [46.7–52.1] | 55.3 [51.3–58.9] | 50.1 [49.0–53.9] |

Continuous data are presented as the medians (interquartile range [IQR]). BMI, body mass index; PNI, prognostic nutritional index; NN, nonsarcopenic/nonobesity; NO, nonsarcopenic/obesity; SN, sarcopenic/nonobesity; SO, sarcopenic/obesity.

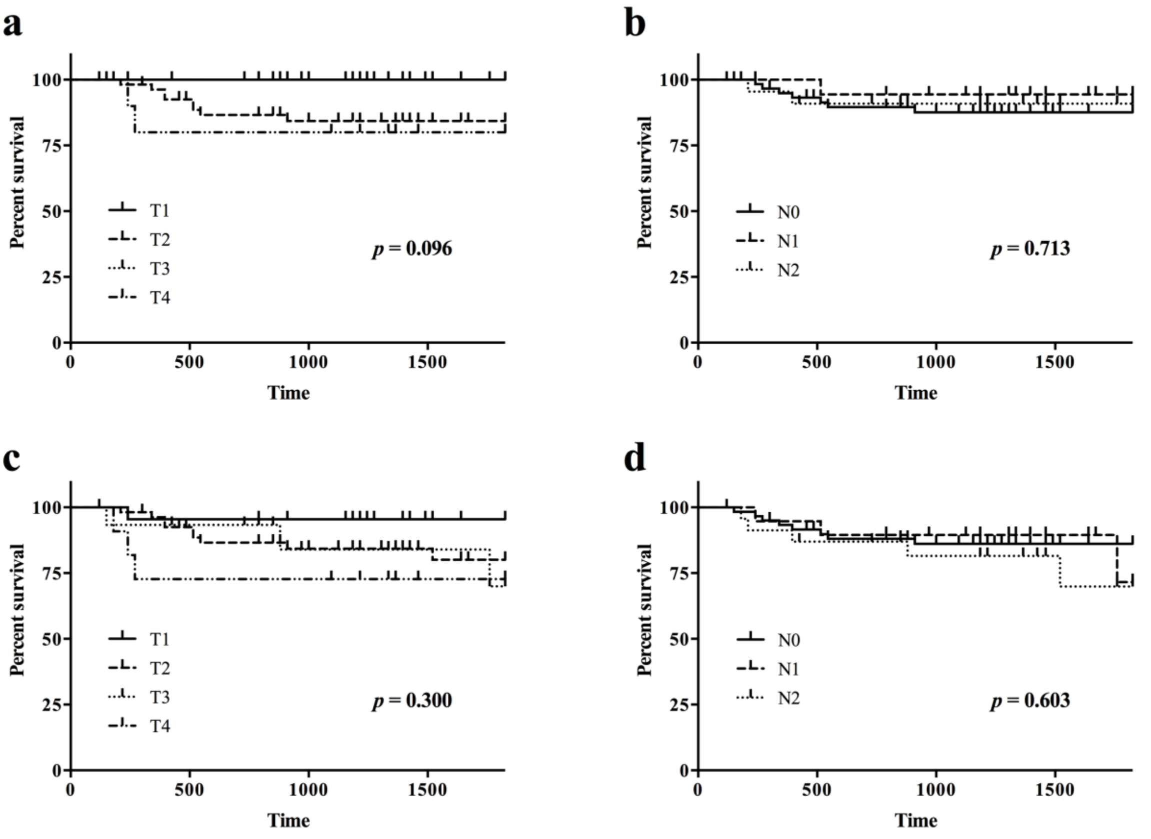


**Figure S2.** Survival rates in patients with OSCC according to T and N classification. There were no statistically significant differences between the groups in DSS (**a**, $p$=0.096; **b**, $p$=0.713) or OS (**c**, $p$=0.300; **d**, $p$=0.603). DSS, disease-specific survival; OS, overall survival.
